# Synthesis of Tropine-Based Functionalized Acidic Ionic Liquids and Catalysis of Esterification

**DOI:** 10.3390/ijms232112877

**Published:** 2022-10-25

**Authors:** Hongfei Ni, Yiwei Zhang, Chuhong Zong, Zhengbo Hou, Hang Song, Yong Chen, Xuesong Liu, Tengfei Xu, Yingjie Luo

**Affiliations:** 1 College of Pharmaceutical Sciences, Zhejiang University, Hangzhou 310058, China; 2 College of Chemical Engineering, Sichuan University, Chengdu 610065, China

**Keywords:** ionic liquids, tropine, catalyst, aspirin, esterification

## Abstract

Some traditional acidic ionic liquids (AILs) have shown great catalytic potential in esterification; meanwhile, the design and application of more new AILs are expected at present.Tropine-based functionalized acidic ionic liquids (FAILs) were synthesized to realize esterification catalysis for the first time; with aspirin synthesis as the template reaction, key influences on the substrate conversion and product yield of the synthesis, such as IL type, ratio of salicylic acid to acetic anhydride, temperature, reaction time and amount of IL, were investigated. The new tropine-based FAILs exhibited excellent performance in catalytic synthesis of aspirin with 88.7% yield and 90.8% selectivity. Multiple recovery and re-usage of N-(3-propanesulfonic acid) tropine is the cation, and *p*-toluenesulfonic acid is the anion. ([Trps][OTs]) shows satisfactory results. When [Trps][OTs] was used to catalyze different esterification reactions, it also showed good results. The above studies proved that ionic liquid [Trps][OTs] could serve as an ideal green solvent for esterification reaction, which serves as a suitable substitute for current catalysts.

## 1. Introduction

Ionic liquids (ILs) are a series of liquid molten salt formed by cations and anions with low saturated vapor pressure, excellent thermal and chemical stability and outstanding electrochemical properties [1], which have been used as solvents with adjustable properties [2,3]. Acidic ionic liquids (AILs) are a class of task-specific ionic liquids having an acidic group in the structure with particular properties [4], and these AILs could be categorized as Lewis [5], Brønsted [6] and B-L dual [7] acidic liquid according to the acidic type of functional group. A common method of preparing Brønsted acidic ionic liquid was introducing an acidic group (such as proton N^+^H, sulfo group -SO_3_H or carboxyl group -COOH) to the anion [8] or employing anions which could release protons in ionization, such as hydrogen sulfate anion HSO_4_^−^, dihydrogen phosphate anion H_2_PO_4_^−^ or another heteropolyacid anion [9]. AILs have been widely studied for their high reactivity similar to liquid acids and convenience in separation from the system like solid bases [10]. A growing number of new ILs with specific function have been synthesized for catalysis [11], separation [12], analysis [13] and material sciences [14]. The esterification reaction is an important reaction type and has a wide range of applications. The reaction rate of the esterification was relatively slow and can be improved by the ILs [15].

Multiple catalysts, including solid super acid [16], heteropoly acid [17], high-acidic cation exchange resin [18] and zeolite molecular sieve [19], have been employed with wide and intensive study in catalysis, yet the mechanism of these catalysts were heterogeneous catalysis with low catalytic activity, together with problems in complexity of synthetic process and high capital cost [20]. Application of ILs in esterification could effectively avoid excess of side reaction and increase productivity [21,22], compared with a conventional inorganic acid catalyst. AILs have both high reactivity and acidity that can be adjusted to a certain extent for different reaction requirements, as the acidity of the ionic liquid can be controlled by changing the length of the substituent and the carbon chain. So, a suitable acidic ionic liquid can be designed as a catalyst for a specific esterification reaction. Some studies [23,24,25] have concentrated on salicylate synthesis with Brønsted-AILs and compounds with both cation and anion, which provide protons with higher acidity and catalytic efficiency than those with only a single acidic ion. Shi et al. [26] employed functional AILs (FAILs) with SO_3_H-functionalized cations and HSO_4_^−^ in salicylic esterification. Among these 6 ILs, [PsPy][HSO_4_] was found to be better than that of non-functionalized ILs. Further studies using AIL in esterification reactions are needed urgently, which will be meaningful for researchers in related fields.

Tropine was a hydrolysate of belladonna alkaloid from henbane or *Atropa belladonna L*. As an extract from natural products, it exhibited different special properties when combined with ionic liquids. It has been reported that the tropine-based ionic liquid had good aqueous solubilization and extraction for curcumin [27] and had good biocompatibility for adsorption of ovalbumin (OVA) [28]. However, there is no report on acidic tropine-based ILs used as catalysts in possible reactions, and both the structure and function for such a IL family need further expansion. If the tropine-based FAILs can be used as one new friendly and effective catalyst to replace those frequently applied inorganic strong acids in esterification, the reaction will accord with the ideas of “green chemistry”. 

In this study, a series of new FAILs with tropine as mother nucleus structure were synthesized and characterized by NMR, FT-IR and UV spectra. The catalytic performances of the FAILs were investigated by using aspirin synthesis as template reaction. The influence of some reaction parameters, such as IL type, ratio of salicylic acid to acetic anhydride, temperature, reaction time and amount of IL were analyzed in the substrate conversion and product yield of the synthesis. The final result was significantly higher than the traditional method [29], N-(3-propanesulfonic acid) tropine was a cation, and p-toluenesulfonic acid was an anion. After multiple recycling, ([Trps][OTs]) shows satisfactory results. In order to investigate the universality of catalytic esterification, [Trps][OTs] was also used to catalyze the synthesis of aromatic acid ester derivatives formed by hydroxyl substitution, showing satisfactory results. The study aimed to not only enrich the current types of FILs, but also expand their current scope of application. It was expected to provide a useful reference for the basic research of green solvents, as well as inspire the synthesis of more new efficient FILs for related researchers. 

## 2. Experimental

### 2.1. Reagents and Materials

All chemical reagents (AR) were bought from Kelong chemical reagents factory (Chengdu, China). The 1H NMR spectra were recorded on an AV-400 NMR spectrometer (Bruker Corporation, Zurich, Switzerland), the sample was prepared in CH_3_OD, tetramethylsilane (TMS) was spiked as internal standard. The UV-Vis spectra of the sample were obtained on a UV-2800 spectrophotometer (Hengping scientific instrument, Shanghai, China) in methanol. The concentrations of the products were quantified by high-performance liquid chromatography (HPLC) using an LC-20AT HPLC instrument (SHIMADZU, Kyoto, Japan). The infrared spectroscopic analysis of the samples was performed on a SPECTRUM 400 FT-IR spectrometer (Perkin-Elmer, Waltham, MA, USA). The concentrations of products were analyzed by a GC 7900 (Techcomp, Chengdu, China) equipped with a TM-1701 column (30 m, 1 μm I.D, 0.32 mm film thickness) and a flame ionization detector (FID).

### 2.2. Acidic Ionic Liquids Synthesized by a Two-Step Process

Firstly, 0.055 mol 1, 3-propane sultone and 0.05 mol tropine was dissolved into 40 mL and 50 mL ethyl acetate, respectively. The liquid mixture was heated and refluxed for 12 h, and then it was filtered to obtain solid white powder after washing with ethyl acetate for several times. The intermediate, N-(3-propanesulfonic acid) tropine salts (Trps) was obtained. Purity was checked by measuring the melting range (88–89 °C) of the product, which was further determined as 98.7% by liquid chromatography (Waters C18 column, 150 × 3.9 mm, 5 μm; CH_3_CN:0.3 M AcOH-water = 1:1; detection wavelength: 210 nm; 1 mL·min^−1^). 

**Figure 1:**
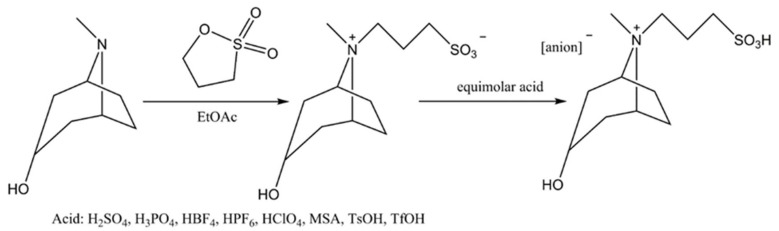
Synthetic route of [Trps][anions].

Then, 0.01 mol Trps was dissolved in 20 mL of de-ionized water, and certain acid of equal molar acid (such as vitriol, phosphoric acid, perchloric acid, fluoboric acid, hexafluorophosphoric acid, methanesulfonic acid, *p*-toluenesulfonic acid and trifluoromethanesulfonic acid) was mixed and stirred for 6 h at room temperature. The target ionic liquid ([Trps][anions]) was obtained after solvent removed. The two-step process above is shown in Figure 1. The structures of products Trps and [Trps][anion] were determined by FT-IR (KBr, 4000~400 cm^−1^) and ^1^H NMR (D_2_O, 600 MHz) analysis.

### 2.3. Acidity Determination of [Trps][Anions]

According to the definition of acid strength, acid strength of Brønsted acidic ionic liquid could be expressed by Hammett acidity function *H*_0_ [30], which is usually determined by UV–Vis spectrophotometry and a basic indicator is used to trap the dissociated proton. This study selected crystal violet as indicator (*pKa* = 0.8) [31] and water as solvent and adopted a UV-2450 spectrophotometer (Shimadzu, Japan) and Hammett indicator method to measure the acidity of ionic liquid solution. Finally, *H*_0_ was obtained according to the Equation (1):

(1) $$H_{0}=pKa+\log([I]/[IH^{+}])$$

where *pKa* is the related value of the indicator (0.99 for tetranitro-aniline), [*I*] and [*IH^+^*] are the molar concentrations of the unprotonated and protonated forms of the indicator, respectively. 

### 2.4. Calculation Method of Catalyze Esterification Reaction Products

**Figure 2:**
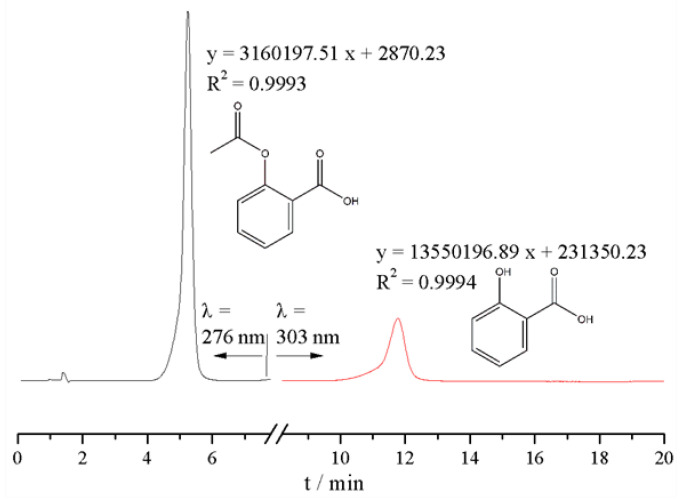
The standard quantitation curves and chromatograms of reaction system.

The reaction process of preparing aspirin was very simple. In this experiment, no other solvent was used as the reaction solvent, and only the substrate salicylic acid, the acylation reagent acetic anhydride and the target ionic liquid as the catalyst were present in the initial reaction system. After the reaction, the reaction solution was analyzed by HPLC to determine the residual amount of substrate and the amount of main product. The analysis conditions were described in reference [32]. In detail, the mobile phase was consistent with CH_3_CN:THF:AcOH:H_2_O = 20:5:5:70 (*V*/*V*/*V*/*V*); the separation was performed on a Waters C18 column (150 × 3.9 mm, 5 µm); flow rate: 1 mL·min^−1^; detection wavelength (λ was 276 nm for aspirin and 303 nm for salicylic acid; sample injection volume was 10 μL and the column temperature was set as 25 °C. The standard operating curves formula and chromatograms of salicylic acid and aspirin are shown in Figure 2.

For more details, some effects on three important objects, i.e., the yield, conversion and selectivity, were investigated. The objects were defined as follow:

(2) $$Yield=\frac{m_{\mathrm{aspirin}(exp.)}}{m_{\mathrm{aspirin}(\mathrm{ideal})}}\times 100\%$$

(3) $$Conversion=1-\frac{m_{SA(\mathrm{residual})}}{m_{SA(\mathrm{initial})}}\times 100\%$$

(4) $$Selectivity=\frac{Yield}{Conversion}\times 100\%$$

Here, salicylic acid (*SA*) conversion, yield and selectivity were calculated from Equations (2)–(4), and various *m_SA_* values were determined by the above HPLC method. 

### 2.5. Application of the New Tropine-Based FILs to Catalyze Esterification Reaction

**Figure 3:**
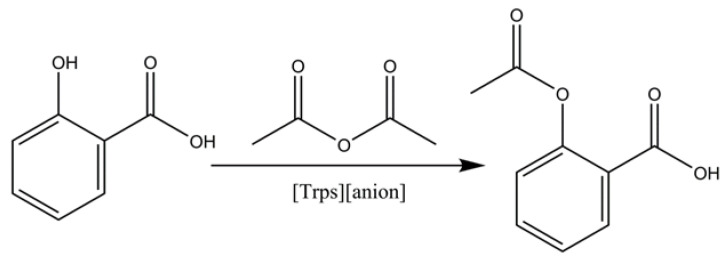
The catalytic synthesis route of aspirin by [Trps][anions].

This experiment design synthesized eight kinds of sulfonic group functionalized ionic liquid to verify the catalysis effect of acid ionic liquid in esterification. Taking aspirin for example, types of ionic liquids, temperature, time, alcohol acid ratio and ionic liquid volume were investigated. The concrete reaction equation is as shown in Figure 3. The specific experimental operation was as follows: 

[Trps][anions] and a certain amount acetic anhydride were added to 0.01 mol salicylic acid and stirred at different temperatures for a period of time to obtain aspirin. After that, aspirin was recovered by extraction with EtOAc with equal volume three times.

### 2.6. Statistical Analysis

Single-factor experiment and response surface methodology (RSM) was applied to obtain the optimization adsorption conditions [33]. Based on single factor research, four factors, namely X_1_ (temperature, °C), X_2_ (time, min), X_3_ (the ratio of acetic anhydride to salicylic acid) and X_4_ (the amount of FAILs, mmol), were studied to determine their influences on the yield of aspirin. The levels in each factor were shown in Table S1. The designed levels of each factor and the whole results of the conducted 29 experiments were shown in Supplemental Material (SM) Table S1 and Table S2, respectively.

### 2.7. Recovery and Reusing Performance of [Trps][OTs]

As a catalyst, it was necessary to be provided with high catalyzing performance and acceptable reusability. The experiment employed isovolumetric distilled water and ethyl acetate respectively to wash gross product of reaction. The water phase was again washed with isovolumetric ethyl acetate, and then a thick liquid, i.e., recovered ionic liquid, was obtained after removing water and acetic acid in the water phase. The recycled ionic liquid was then repeated to obtain the performance data.

### 2.8. Synthesis of Different Ester Compounds Catalyzed by [Trps][OTs]

The acetylation reactions of *p*-hydroxybenzoic acid and *m*-hydroxybenzoic acid were catalyzed by ionic liquid [Trps][OTs] for further investigating the catalyzing performance of [Trps][OTs]. The reaction and analysis conditions were the same as those of previous salicylic acid acetylation. When ionic liquid catalyzed benorilate, the input amount of the two raw materials was all 0.01 mol, and the dosage of catalyst [Trps][OTs] was 1 mmol at 100 °C. Analysis conditions of salicylic acid alcohol ester: mobile phase CH_3_OH:1% AcOH (a.q.) = 80:20; Diamonsil 5 μm C18 chromatographic column, 250 × 4.6 mm; flow rate = 1.0 mL × min^−1^; detection wavelength = 254 nm; column temperature = 25 °C. 

## 3. Results and Discussions 

### 3.1. Preparation of Trps and [Trps][Anions]

As shown in Figure S1 and SM, the selected eight anions included hydrogen sulfate [HSO_4_], methanesulfonic acid [OMs], *p*-toluenesulfonic acid [OTs], trifluoromethanesulfonic acid [OTf], dihydrogen phosphate [H_2_PO_4_], perchloric acid [ClO_4_], fluoboric acid [BF_4_] and hexafluorophosphoric acid [PF_6_], respectively. The yields of the eight ionic liquids were all above 95%. The ionic liquids looked achromic or like light brown transparent thick liquid in Figure S1, and were stored in vacuum and dry conditions before use. 

### 3.2. Structural Identification and Spectral Analysis of Trps and [Trps][Anions]

**Figure 4:**
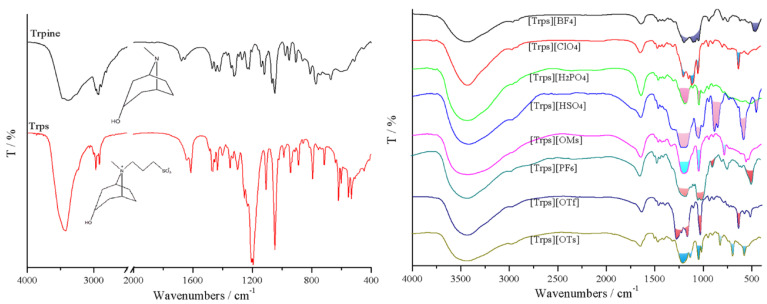
FT-IR spectra of Tropine, Trps and [Trps][anion].

Tropine, Trps and eight target ionic liquids were verified via infrared spectra in Figure 4. Detailed infrared analysis of tropine, Trps and [Trps][OTs] were shown in Table S2. The difference among the eight ionic liquid IR spectra was mainly embodied in anion absorption peak.

**Figure 5:**
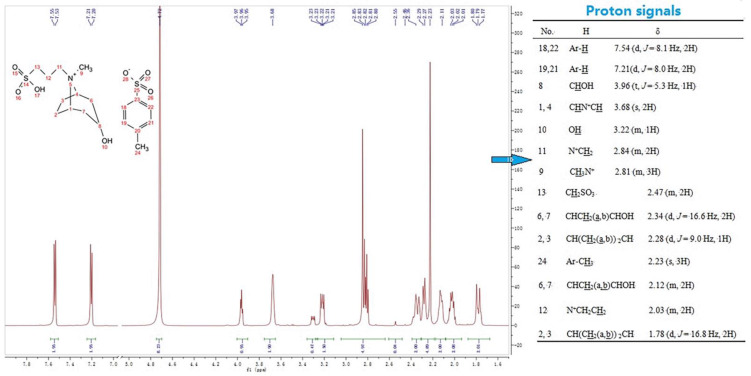
^1^H NMR spectra of [Trps][OTs] (D_2_O, 400 MHz).

The ^1^H NMR spectra of [Trps][OTs] is shown in Figure 5, and the detailed hydrogen spectrum data of the eight types of ionic liquids synthesized are shown in Table S3 (see SM). When the anion was changed, the chemical shift of H atoms (H-1–9) in cation varied in the range of 0.1–0.2 ppm, and the main contribution for the spectral difference results from the proton signals (protons on acid group and benzene ring) of anions. Taking the data of [Trps][OTs] as an example for analysis, H-8 directly connected to O can be found in the lower-field regime (3.96, 1H); the quaternarized N atom carries a positive charge to produce a deshielding effect on surrounding H atoms in a manner. The hydrogen spectrum of [Trps][OTs] shows two groups of characteristic H signals at the ortho (7.54, 2H) and meta (7.21, 2H) positions of the sulfonic acid group on the aromatic ring in the anion; additionally, there was a CH_3_ peak on the aromatic ring at 2.23 ppm. Introducing of anions made resonance signals of cation protons generally move toward a higher field by comparison with those of Trps. 

### 3.3. Hammett Acidity Analysis of [Trps][Anions]

**Figure 6:**
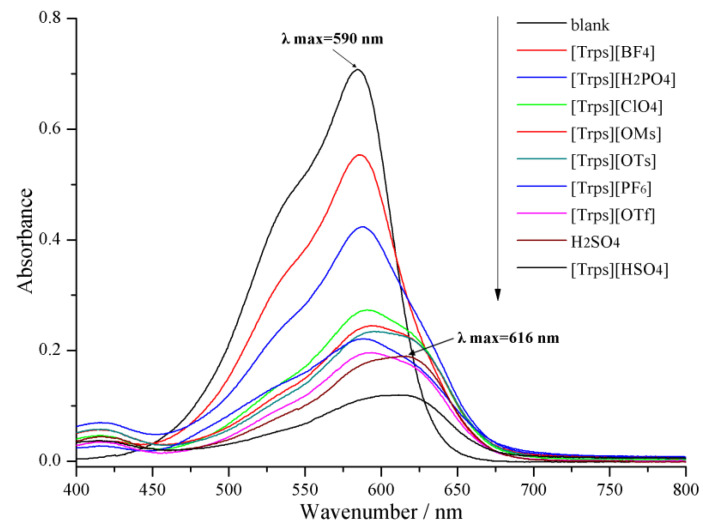
Absorbance spectra of 0.01 g·L^−1^ crystal violet for various 25 mmol·L^−1^ [Trps][anion].

Crystal violet was selected as the indicator, and the spectrum scan graph for measured ionic liquid and vitriol with equal molar concentration was shown in Figure 6. The weakening degree of maximal absorbance of 0.01 g L^−1^ crystal violet was varying due to different binding capacities of different ionic liquids with indicator. When hydrogen sulfate was the anion, the maximal absorbance of indicator reduces by 83.05%, and Hammett value was 0.1098 under a concentration of 25 mmol L^−1^, which shows the strongest acidity. 

With increasing acidity of the acidic ionic liquid, the concentration of the unprotonated form of the basic indicator decreases, whereas the protonated form of the indicator could not be observed because of its weak absorbance and its location; so, the [I]/[IH^+^] ratio could be determined from the measured absorbance differences after addition of new acidic ionic liquid. It was observed that from the Hammett acidity result in Table S4 (see SM), the acidity of ionic liquid was largely related to anion. In the water system, protons were ionized out from the sulfonic group and can be recombined with sulfonic acid of the IL cation of and IL anions (e.g., H_2_PO_4_^−^, HSO_4_^−^, ClO_4_^−^, OMs^−^, OTs^−^). The proton concentration was determined by the ionization equilibrium of these two groups in ionic liquid aqueous solution. Higher proton concentration could lead to stronger acidity. The acidities of eight ILs and H_2_SO_4_ increase as follows: [Trps][BF_4_] < [Trps][H_2_PO_4_] < [Trps][ClO_4_] < [Trps][OMs] < [Trps][OTs] < [Trps][PF_6_] < [Trps][HSO_4_] < [Trps][OTf] < H_2_SO_4_ < [Trps][HSO_4_]. Moreover, the acidity of these ILs is much stronger than that of the reported benzothiazolium ILs with similar acidic anions (*H*_0_ in 1.10–1.46) except [Trps][BF_4_] [34]. The stronger acidity of the ionic liquid could be more apt to dissociate out protons in the ionization equilibrium. As an anion, HSO_4_ could also further ionize out protons to raise the proton concentration. Hence, the acidity of [Trps][HSO_4_] was the strongest. 

### 3.4. Effect of Ionic Liquids on the Reaction 

**Figure 7:**
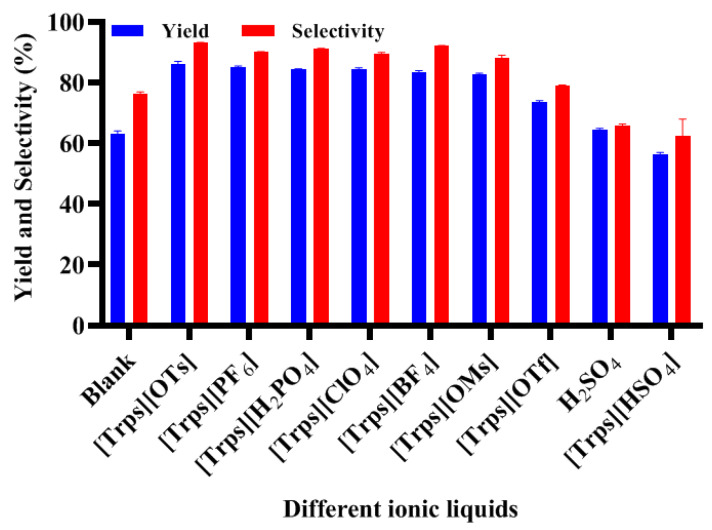
Effect of FILs structures on the esterification reaction results.

The results catalyzed by different ILs in the same condition (0.01 mol salicylic acid, 0.02 mol acetic anhydride, 1 mmol ionic liquid, at 90 °C, for 40 min) are shown in Figure 7. A blank experiment without catalyst and a reaction catalyzed by H_2_SO_4_ were carried out in the same conditions for distinguishing contribution from the ILs.

Basically, the conversion increased with higher acidity of FILs and is coupled with increase of side reaction, which would decrease the selectivity of reaction and the yield of target product. Some other factors will also affect the reaction results, such as ionic liquid viscosity and ionic liquid dissolving ability for reactants; so, acidity is analyzed as the main reason here. Here, the ionic liquid viscosity was measured using an NDJ-1 type viscosity meter (DECCA Inc., Shanghai, China) at room temperature, which shows the results of 243 cP ([Trps][BF_4_]), 323 cP ([Trps][H_2_PO_4_]), 264 cp ([Trps][ClO_4_]), 277 cP ([Trps][OMs]), 201 cp ([Trps][OTs]), 309 cP ([Trps][PF_6_]), 342 cP ([Trps][OTf]) and 396 cP ([Trps][HSO_4_]). In a homogeneous system under heating conditions, the collision probability of substrate and acylating reagent molecule could increase, so the conversions for blank reaction and for H_2_SO_4_-catalyzed reaction could achieve 62% and 73%. However, their reaction yield and selectivity were apparently lower compared with ones catalyzed with even weak acidity of ionic liquid. Comprehensively considering product yield, reaction selectivity and ionic liquid preparation, [Trps][OTs] is suggested as the optimum catalyst for aspirin reaction. Generally, the cations of acidic ILs play an important role in their selectivity and solubility, while the anions reflecting the difference among IL acidity have an obvious impact on the yield when the ILs have the same cation, so the catalytic performance is often the result of the coordination of anions and cations. It should also be worth mentioning that no extra solvent and catalyst is needed in the esterification of this study, and the selected IL can play the dual roles of solvent and catalyst. Differently, cyclohexane (co-solvent) or HCl (co-catalyst) were necessary in the reported processes catalyzed by acidic ILs or silica-immobilized acidic ILs before [35,36]. 

### 3.5. Effects of Temperature, Reaction Time, Molar Ratio of Alcohol and Acid Anhydride and Amount of Ionic Liquid on Reaction

**Figure 8:**
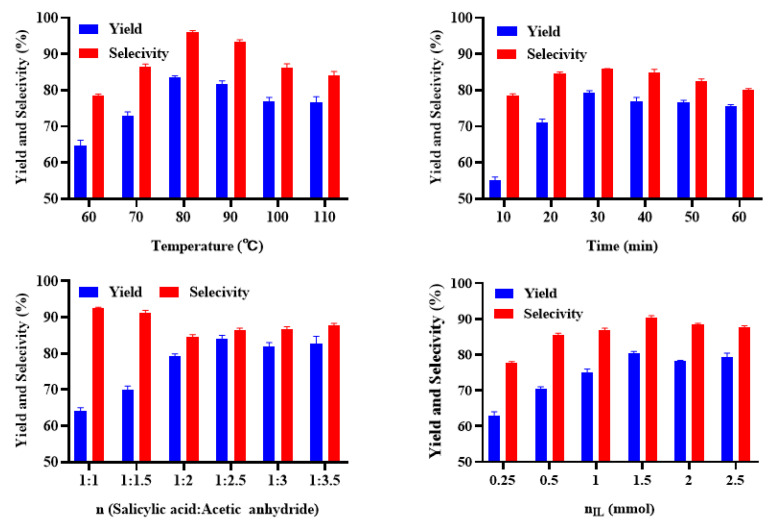
Effect of temperature, time, ratio of salicylic acid to acetic anhydride and amount of [Trps][OTs] on the esterification reaction results.

Based on the above results, [Trps][OTs] has shown ideal performance both in yield and selectivity among the candidates. Here, catalysis by this kind of catalyst was illustrated to the effects, and the results are shown in Figure 8. As reaction temperature rose, the conversion of substrate increased gradually. However, too high temperature could lead to increase of side reaction and thereby to decrease of reaction selectivity and product yield. A better reaction temperature was 80 °C with 85.35% of product yield and 92.84% of selectivity. 

The effects of reaction time, ratio of salicylic acid to acetic anhydride and amount of ILs were investigated at 80 °C. The conversion of substrate and yield of target product or reaction selectivity increase with longer reaction time, higher ratio and larger amount, but the yield or selectivity trended to a peak at a certain point. In comparison, the reaction speed catalyzed by this applied acidic IL is obviously faster than 1-methylimidazolium tetrafluoroborate (2~10 h) [37], and its consumption is much lower than that in previous study [38]. 

### 3.6. RSM Optimization

To explore the correlation of mentioned experimental factors and obtain the maximum yield of aspirin, RSM was further applied to optimize the reaction conditions. The results of variance analysis and credibility analysis of this regression model were shown in Table 1. Among them, F and P value were used to judge the significance of the model and various variables to the response results, respectively. Based on the four experimental factors, the quadratic regression model was significant (F = 1.449 × 10^6^, *p* < 0.0001), and the loss of fit term was not significant (F = 0.083, *p* = 0.9992), indicating that the model can be used to predict the catalytic synthesis of aspirin. The quadratic regression model between yield and the four selected factors met the following equation:

S = 80.86 + 6.57X_1_ + 11.11X_2_ + 9.09X_3_ + 4.55X_4_ − (5.000 × 10^−3^)X_1_X_2_ − (2.500 × 10^−3^)X_1_X_3_ + (7.500 × 10^−3^)X_1_X_4_ − (5.000 × 10^−3^)X_3_X_4_ − 11.64X_1_^2^ − 13.16X_2_^2^ − 6.09X_3_^2^ + 0.48X_4_^2^,

in which, S, X_1_, X_2_, X_3_ and X_4_ represents the yield of aspirin, reaction temperature, time, the ratio of salicylic acid to acetic anhydride and the amount of FAILs, respectively. The reliability analysis of this model showed that the coefficient of variation (CV%) of the experiment was 0.023%, and the standard deviation was 0.016, both within the acceptable range, indicating that the repeatability of the data in each separate experiment was good. Meanwhile, the R^2^ of the regression model was 0.9998, which also indicated that the correlation degree between the points of experimental and predicted complies with a good regression model. The difference between the adjusted R^2^ value and the predicted R^2^ value was far less than 0.01, which also indicated the high reliability of the proposed prediction model. 

According to the RSM results, the optimal predicted crystallization conditions include: reaction temperature at 87.8 °C, reaction time at 35.3 min and the ratio of salicylic acid to acetic anhydride at 1:3. The amount of ionic liquid at 1.5 mmol, with the predicted yield of aspirin can reach 89.3%. Three further experimental verifications were carried out under these fixed conditions. Finally, the yield of aspirin reached 88.7%, and based on our best knowledge, the yield was significantly higher than that reported in the literature [29].

### 3.7. Recovery and Reusing Performance of [Trps][OTs]

**Figure 9:**
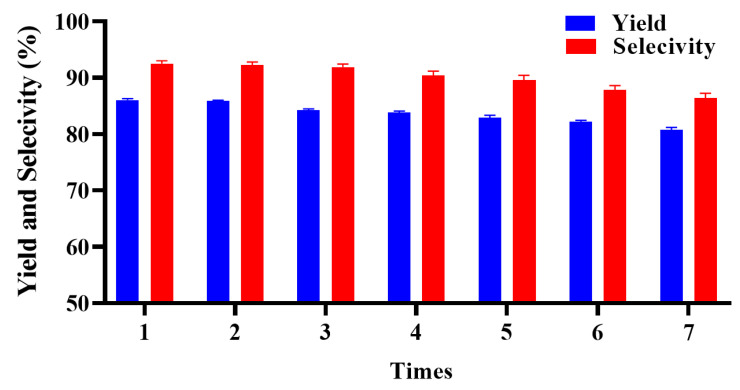
Reusability of [Trps][OTs] by 7 cycles.

The results of the recovery test are shown in Figure 9. The conversion of substrate, product yield and reaction selectivity fluctuated around the initial catalyzing effect or appeared to slightly drop. For instance, the reaction yield after the fifth recovering was kept to 81.1%, which is very close to the first yield of 88.7%. Once the yield and selectivity drop to an unacceptable level for users, a more efficient resin purification method is recommended instead of the simple washing method [38,39]. 

### 3.8. Synthesis of Different Ester Compounds Catalyzed by [Trps][OTs] 

The conversion of salicylic acid (0.01 mol salicylic acid and 0.01 mol alcoholic hydroxyl solution reacted for a certain time at 100 °C in 1.5mmol [Trps][OTs] catalyst) was shown in Table 2. The acetylation reaction of hydroxyl catalyzed by [Trps][OTs] obtained good result. As shown in Table 2, the yield of the product could reach 90% or more in a short period of time, which proved that the [Trps][OTs] had good applicability in catalyzing esterification reactions.

## 4. Conclusions

In conclusion, eight new functionalized acid ILs with sulfonic groups were synthesized, and the Hammett acidity intensity of the ILs in concentration 25 mol·L^−1^ follows as [Trps][BF_4_] < [Trps][H_2_PO_4_] < [Trps][ClO_4_] < [Trps][OMs] < [Trps][OTs] < [Trps][PF_6_] < [Trps][HSO_4_] < [Trps][OTf]. The aspirin synthesis catalyzed by [Trps][anions] demonstrates good catalyzing performance. Among the [Trps][anions], [Trps][OTs] presented the best with 88.7% yield and 90.8% selectivity. In addition, the ionic liquid [Trps][OTs] has satisfactory reusability, which shows promising good environmental and economic application. In addition to the previous aspirin synthesis, the synthesizing reactions of a series of esterification products of salicylic acid catalyzed by [Trps][OTs] further demonstrate that ionic liquid [Trps][OTs] could serve as an ideal catalyst for esterification reaction. To sum up, these new FILs not only enrich the current types of ILs, but also expand the current scope of application. The results obtained can not only provide a useful reference for the basic research of green chemical mediums, but also inspire the synthesis of more new efficient functional ILs. The method established in this study can balance selectivity and yield, while the time is shorter than that in some esterification studies and less catalyst is used through comparison. 

## Figures and Tables

**Table 1:** Results of the variance analysis from the response surface test.

Source	Sum of Squares	d f	Mean Square	F Value	*p*-Value Prob > F	
Model	5113.06	14	365.22	1.45 × 10^6^	<0.0001	significant
X_1_	517.45	1	517.45	2.05 × 10^6^	<0.0001	
X_2_	1481.63	1	1481.63	5.88 × 10^6^	<0.0001	
X_3_	990.99	1	990.99	3.93 × 10^6^	<0.0001	
X_4_	248.34	1	248.34	9.85 × 10^5^	<0.0001	
X_1_X_2_	1.00 × 10^−4^	1	1.00 × 10^−4^	0.40	0.5389	
X_1_X_3_	2.50 × 10^−5^	1	2.50 × 10^−5^	0.10	0.7574	
X_1_X_4_	2.25 × 10^−4^	1	2.25 × 10^−4^	0.89	0.3607	
X_2_X_3_	0.00	1	0.000	0.00	1.0000	
X_2_X_4_	0.00	1	0.000	0.00	1.0000	
X_3_X_4_	1.00 × 10^−4^	1	1.00 × 10^−4^	0.40	0.5389	
X_1_^2^	878.68	1	878.68	3.49 × 10^6^	<0.0001	
X_2_^2^	1123.17	1	1123.17	4.46 × 10^6^	<0.0001	
X_3_^2^	240.38	1	240.38	9.54 × 10^5^	<0.0001	
X_4_^2^	1.49	1	1.49	5927.88	<0.0001	
Lack of Fit	6.08 × 10^−4^	10	6.08 × 10^−5^	0.083	0.9992	not significant
Pure Error	2.92 × 10^−3^	4	7.30 × 10^−4^			
Cor Total	5113.06	28				

**Table 2:** The esterification of hydroxy-substituted benzoic acid catalytic synthesized by [Trps][OTs].

No.	Component (A)	Component (B)	C (Target Product)	Reaction Time/min	Conversion/%
1	*p*-hydroxybenzoic acid	Acetic anhydride	*p*-acetoxybenzoic acid	4.469	95.13
2	*m*-hydroxybenzoic acid	Acetic anhydride	*m*-acetoxybenzoic acid	4.448	88.57
3	Acetylsalicylic acid	Acetaminophen	Benorilate	7.893	84.22
4	Salicylic acid	Methanol	Methyl salicylate	5.653	97.35
5	Salicylic acid	Alcohol	Ethyl salicylate	7.424	97.54
6	Salicylic acid	Propyl alcohol	Propyl salicylate	14.699	98.13
8	Salicylic acid	Butyl alcohol	Butyl salicylate	14.709	96.83

## Supplementary Materials

The following supporting information can be downloaded at: https://www.mdpi.com/article/10.3390/ijms232112877/s1.
