# Supplementary material for: Synthesis of Tropine-Based Functionalized Acidic Ionic Liquids and Catalysis of Esterification
Source: Int J Mol Sci. 2022 Oct 25;23(21):12877. doi: 10.3390/ijms232112877 (PMC9656471; doi:10.3390/ijms232112877)
Supplement: Supplementary file 1 [file ijms-23-12877-s001.zip › ijms-1946511-supplementary.pdf]

Supplemental Material

Synthesis of Tropine-Based Functionalized Acidic Ionic Liquids and Catalysis of Esterification

Table of content

Figure S1 **page 3**

Table S1 **page 4**

Table S2 **page 5**

Table S3 **page 6**

Table S4 **page 7**

Table S5 **page 8**

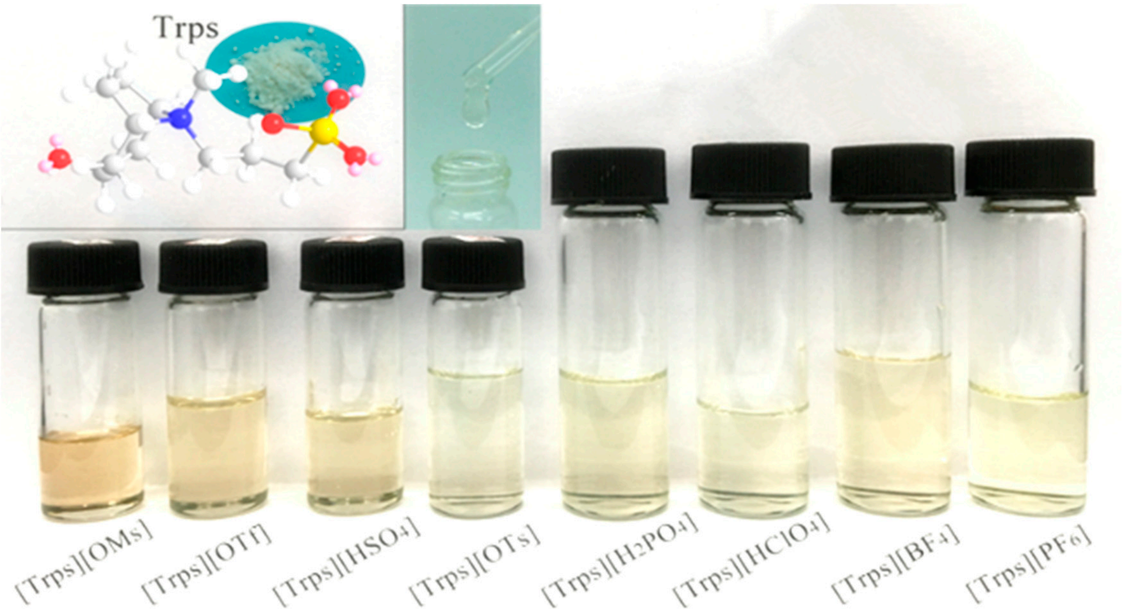

Figure S1. The photos of [Trps][anion]

Table S1. Test factor level and coding table of the response surface method.

Factor	Levels		
	-1	0	1
X_1	60	80	100
X_2	10	30	50
X_3	1	2	3
X_4	0.5	1	1.5

Table S2. The test design table and results of response surface method analysis.

Number	X1	X2	X3	X4	Yield(%)
1	0	0	-1	1	70.72
2	1	0	0	-1	71.71
3	-1	-1	0	0	38.38
4	1	-1	0	0	51.51
5	-1	0	1	0	65.65
6	0	1	1	0	81.81
7	0	0	0	0	80.82
8	-1	0	0	1	67.67
9	0	0	-1	-1	61.61
10	0	-1	-1	0	41.41
11	0	0	1	1	88.88
12	0	0	0	0	80.84
13	1	0	1	0	78.78
14	-1	1	0	0	60.62
15	1	0	0	1	80.83
16	0	1	0	1	83.83
17	0	1	0	-1	74.74
18	1	0	-1	0	60.61

19	-1	0	-1	0	47.47
20	0	-1	0	1	61.61
21	1	1	0	0	73.73
22	0	0	0	0	80.86
23	-1	0	0	-1	58.58
24	0	0	0	0	80.87
25	0	0	0	0	80.89
26	0	-1	1	0	59.59
27	0	1	-1	0	63.63
28	0	0	1	-1	79.79
29	0	-1	0	-1	52.52

Table S3. FT-IR spectra analysis of Tropine, Trps and [Trps][OTs]

The functional group vibrates	Wave(cm^{-1})	The functional group vibrates	Wave(cm^{-1})
		Trpine	
-OH	3390	C-N Asymmetric stretching vibration	1471, 1425
N-CH_3，-CH_2-	2980~2880	C-N Symmetric stretching vibration	1321,1225
Vibration of bridge ring frame	1678, 1654	C-O	1065
		Trps	
-OH	3419	SO_2	1299
-CH_3	2963	S=O	1205, 1195
-CH_2-	2909	C-S-O	1108, 944
-CH_2, -CH_3	1471,1452	-（CH_2）-	715~800
C-N^+	1351, 1254	-SO_3^-	621, 534
		[Trps][OTs]	
-OH	3430	S=O	1126
-CH_3，-CH_2-	2850~3000	CHOH	1040
Vibration of bridge ring frame	1640, 1494	C-S-O	1011, 942
-CH_2, -CH_3	1461,1437	Ar-H	818
C-N^+	1399, 1310	-（CH_2）-	689
SO_2	1206	-SO_3	616, 569

Table S4. NMR data of ILs.

IL	Frequency	Solvent	Chemical shift (ppm) and peak attribution
Trps	400 MHz	D_2O	δ: 4.11 (t, J = 5.6 Hz, 1H), 3.86 (s, 2H), 3.46 (m, 1H), 3.37 (m, 2H), 2.99 (m, 3H), 2.93 (dd, J = 15.1, 7.9 Hz, 2H), 2.50 (d, J = 16.4 Hz, 2H), 2.42 (d, J=8.8 Hz, 2H), 2.28 (m, 2H), 2.16 (m, 2H), 1.92 (d, J = 17.0 Hz, 2H)
[Trps][OTs]	400 MHz	D_2O	δ: 7.54 (d, J = 8.1 Hz, 2H), 7.21(d, J = 8.0 Hz, 2H), 3.96 (t, J = 5.3 Hz, 1H), 3.68 (s, 2H), 3.22 (m, 1H), 2.84 (m, 2H), 2.81 (m, 3H) 2.47 (m, 2H), 2.34 (d, J = 16.6 Hz, 2H), 2.28 (d, J = 9.0 Hz, 1H), 2.23 (s, 3H), 2.12 (m, 2H), 2.03 (m, 2H), 1.78 (d, J = 16.8 Hz, 2H)
[Trps][OMs]	400 MHz	D_2O	δ: 3.91 (t, J = 5.1 Hz, 1H), 3.79 (s, 2H), 3.41 (m, 1H), 3.33 (m, 2H), 2.92 (m, 3H), 2.85 (dd, J = 14.8, 7.5 Hz, 2H), 2.56(s, 3H), 2.46 (d, J = 15.8 Hz, 2H), 2.38 (d, J=8.3 Hz, 2H), 2.22 (m, 2H), 2.12 (m, 2H), 1.83 (d, J = 16.2 Hz, 2H)

[Trps][OTf]	400 MHz	D_2O	δ: 4.16 (t, J = 5.8 Hz, 1H), 3.91 (s, 2H), 3.52 (m, 1H), 3.43 (m, 2H), 3.03 (m, 3H), 2.97 (dd, J = 15.3, 8.2 Hz, 2H), 2.56 (d, J = 16.9 Hz, 2H), 2.47 (d, J=9.3 Hz, 2H), 2.33 (m, 2H), 2.26 (m, 2H), 1.96 (d, J = 17.6 Hz, 2H)
[Trps][HSO_4]	400 MHz	D_2O	δ 12.92 (s, 1H), 4.01 (t, J = 5.3 Hz, 1H), 3.79 (s, 2H), 3.38 (m, 1H), 3.26 (m, 2H), 2.88 (m, 3H), 2.83 (dd, J = 14.8, 7.6 Hz, 2H), 2.56 (d, J = 15.8 Hz, 2H), 2.38 (d, J=8.2 Hz, 2H), 2.24 (m, 2H), 2.08 (m, 2H), 1.88 (d, J = 16.3 Hz, 2H)
[Trps][H_2PO_4]	400 MHz	D_2O	δ: 11.11(s, 2H), 3.96 (t, J = 5.3 Hz, 1H), 3.66 (s, 2H), 3.38 (m, 1H), 3.29 (m, 2H), 2.89 (m, 3H), 2.78 (dd, J = 13.8, 6.9 Hz, 2H), 2.41 (d, J = 15.9 Hz, 2H), 2.32 (d, J=7.9 Hz, 2H), 2.17 (m, 2H), 2.09(m, 2H), 1.79 (d, J = 16.7 Hz, 2H)
[Trps][ClO_4]	400 MHz	D_2O	δ: 3.91 (t, J = 5.39 Hz, 1H), 3.77 (s, 2H), 3.35(m, 1H), 3.24 (m, 2H), 2.83 (m, 3H), 2.75 (dd, J = 14.2, 6.4 Hz, 2H), 2.32 (d, J = 15.9 Hz, 2H), 2.29 (d, J=7.5 Hz, 2H), 2.18 (m, 2H), 2.08 (m, 2H), 1.82 (d, J = 16.1 Hz, 2H)
[Trps][BF_4]	400 MHz	D_2O	δ: 3.89 (t, J = 5.52 Hz, 1H), 3.79 (s, 2H), 3.41 (m, 1H), 3.39 (m, 2H), 3.02 (m, 3H), 2.98 (dd, J = 15.4, 8.1 Hz, 2H), 2.55 (d, J = 16.8 Hz, 2H), 2.47 (d, J=9.1 Hz, 2H), 2.31 (m, 2H), 2.26 (m, 2H), 1.98 (d, J = 17.3 Hz, 2H)
[Trps][PF_6]	400 MHz	D_2O	δ: 4.16 (t, J = 5.8 Hz, 1H), 3.93 (s, 2H), 3.56 (m, 1H), 3.44 (m, 2H), 3.08 (m, 3H), 3.12 (dd, J = 15.8, 8.2 Hz, 2H), 2.56 (d, J = 17.1 Hz, 2H), 2.51 (d, J=9.2 Hz, 2H), 2.38 (m, 2H), 2.27 (m, 2H), 2.01 (d, J = 17.8 Hz, 2H)

Table S5. Calculation and comparison of H_0 values for 25 mmol·L^{-1} [Trps][anion] with 0.01 g·L^{-1} crystal violet at 20A.

ILs	A_{max}	[*I*](%)	[*I*](%)	H_0
blank	0.7080	100	-	-
[Trps][BF_4]	0.5540	78.25	21.75	1.3560
[Trps][H_2PO_4]	0.4230	59.75	40.25	0.9715
[Trps][ClO_4]	0.2732	38.59	61.41	0.5983
[Trps][OMs]	0.2453	34.65	65.35	0.5244
[Trps][OTs]	0.2343	33.09	66.91	0.4942
[Trps][PF_6]	0.2210	31.21	68.79	0.4569
[Trps][OTf]	0.1962	27.71	72.29	0.3836
H_2SO_4	0.1900	26.84	73.16	0.3644
[Trps][HSO_4]	0.1200	16.95	83.05	0.1098
